# How landscape scale changes affect ecological processes in conservation areas: external factors influence land use by zebra (*Equus burchelli*) in the Okavango Delta

**DOI:** 10.1002/ece3.676

**Published:** 2013-07-22

**Authors:** Hattie L A Bartlam-Brooks, Mpaphi C Bonyongo, Stephen Harris

**Affiliations:** 1 School of Biological Sciences, University of BristolWoodland Road, Bristol, BS8 1UG, United Kingdom; 2 Okavango Research Institute, University of BotswanaPrivate Bag 285, Maun, Botswana

**Keywords:** Climate change, equids, foraging efficiency, GPS collars, habitat patches, kernel density estimates, landscape ecology, RAMSAR, resource utilization functions, wetland management

## Abstract

Most large-bodied wildlife populations in sub-Saharan Africa only survive in conservation areas, but are continuing to decline because external changes influence ecological processes within reserves, leading to a lack of functionality. However, failure to understand how landscape scale changes influence ecological processes limits our ability to manage protected areas. We used GPS movement data to calculate dry season home ranges for 14 zebra mares in the Okavango Delta and investigated the effects of a range of landscape characteristics (number of habitat patches, mean patch shape, mean index of juxtaposition, and interspersion) on home range size. Resource utilization functions (RUF) were calculated to investigate how specific landscape characteristics affected space use. Space use by all zebra was clustered. In the wetter (Central) parts of the Delta home range size was negatively correlated with the density of habitat patches, more complex patch shapes, low juxtaposition of habitats and an increased availability of floodplain and grassland habitats. In the drier (Peripheral) parts of the Delta, higher use by zebra was also associated with a greater availability of floodplain and grassland habitats, but a lower density of patches and simpler patch shapes. The most important landscape characteristic was not consistent between zebra within the same area of the Delta, suggesting that no single foraging strategy is substantially superior to others, and so animals using different foraging strategies may all thrive. The distribution and complexity of habitat patches are crucial in determining space use by zebra. The extent and duration of seasonal flooding is the principal process affecting habitat patch characteristics in the Okavango Delta, particularly the availability of floodplains, which are the habitat at greatest risk from climate change and anthropogenic disturbance to the Okavango's catchment basin. Understanding how the factors that determine habitat complexity may change in the future is critical to the conservation of large mammal populations. Our study shows the importance of maintaining flood levels in the Okavango Delta and how the loss of seasonal floodplains will be compounded by changes in habitat configuration, forcing zebra to change their relative space use and enlarge home ranges, leading to increased competition for key resources and population declines.

## Introduction

Most large-bodied wildlife populations in sub-Saharan Africa only survive in spatially contained protected regions (Newmark 1996). However, many are still declining because external changes influence ecological processes within conservation areas, leading to a lack of functionality (Western et al. 2009; Fynn and Bonyongo 2010). Wetland ecosystems are particularly vulnerable to external land use changes (Turner et al. 2000). The Okavango Delta, an inland wetland covering 15,000 km^2^ in north-west Botswana, southern Africa, is a RAMSAR site with high densities of large herbivores (Bonyongo and Harris 2007) that typifies many of these problems.

The Okavango Delta's catchment basin covers circa 350,000 km^2^ across Angola, Botswana and Namibia (Mbaiwa 2004), making sustainable management of the entire basin practically and politically complicated. The Okavango Delta floods annually, with flood water arriving in the north-west in March and reaching the south-eastern Delta by July (McCarthy and Ellery 1998). The duration of inundation and extent of the seasonal floodplains vary with local topography, and amount and progression of the flood water (Mendelsohn et al. 2010). However, currently proposed water extraction and hydroelectric schemes outside Botswana would, if implemented, have a significant impact on water flow into the Delta. An estimated 95 km^2^ of wetland, mainly seasonal floodplains, would be lost for every 100 million m^3^ of water extracted (Gumbricht et al. 2004). Climatic variation also plays an important role in yearly flood variation (Gumbricht et al. 2004), and climate change is likely to lead to increased climatic variability and reduced rainfall (Hulme et al. 2001), potentially decreasing Okavango flood levels (Wolski and Murray-Hudson 2008). While the combined effects of climatic variation and anthropogenic disturbances could lead to significant loss of seasonal wetlands in the Okavango Delta, how such landscape scale changes influence ecological processes is poorly understood, and so limits our ability to manage protected areas (Hansen and DeFries 2007).

Flooding variation is one of the principal factors driving habitat heterogeneity in the Okavango Delta (McCarthy et al. 2000), creating a temporally and spatially shifting mosaic of habitat patches across the landscape. Despite little change in elevation and a homogeneous sand substrate, the Okavango Delta is a highly heterogeneous, dynamic system, with substantial small-scale spatial variation in vegetation structure dependent on flooding characteristics (Ramberg et al. 2006; Bartlam 2010). Variation in the density and distribution of key resources within a landscape influence animal distribution (Whittaker and Levin 1977), and the shape and complexity of habitat patches and their relative juxtaposition and fragmentation within a landscape influence the shape and size of home ranges (e.g., Tufto et al. 1996; Anderson et al. 2005) and animal movements (Wheatley and Johnson 2009).

We investigated how landscape heterogeneity and the potential impact of external management decisions and climate change influence space use by zebra (*Equus burchelli*) in the Okavango Delta. Because of their hindgut digestive system, equids require high forage consumption to meet their nutritional requirements (Duncan et al. 1990; Menard et al. 2002), and so zebra prefer habitats with a high homogeneous availability of grass and high forage biomass (Gwynne and Bell 1968; Voeten and Prins 1999) where intake rate can be maximized (Spalinger and Hobbs 1992) and foraging efficiency improved (Bergman et al. 2000; Hobbs et al. 2003). Therefore, we hypothesized that resource availability and landscape heterogeneity were important in determining zebra home range size and shape, with zebra having smaller home ranges in the wetter Central Delta, where the availability of preferred open foraging habitats is greater, than in the drier Peripheral Delta. We also hypothesized that space use, as defined by resource utilization functions (RUFs), would be positively related in both regions to (a) smaller patch size and thus increasing habitat patch density, (b) increasing availability of preferred open foraging habitats, and (c) more homogeneous distribution of similar habitat patches.

Our study has quantified how changing flood levels in the Okavango Delta due to proposed water extraction schemes and climate change will lead to the loss of seasonal floodplains and changes in habitat configuration, and how this will drive zebra, a bulk grazer, to use larger home ranges. This and the associated increased competition for key resources will lead to population declines.

## Methods

### Study area

**Figure 1:**
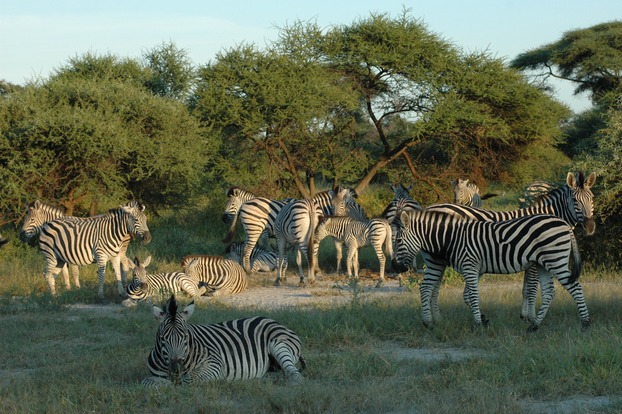
Zebra resting in Acacia woodland in the Moremi Game Reserve, Okavango Delta.

**Figure 2:**
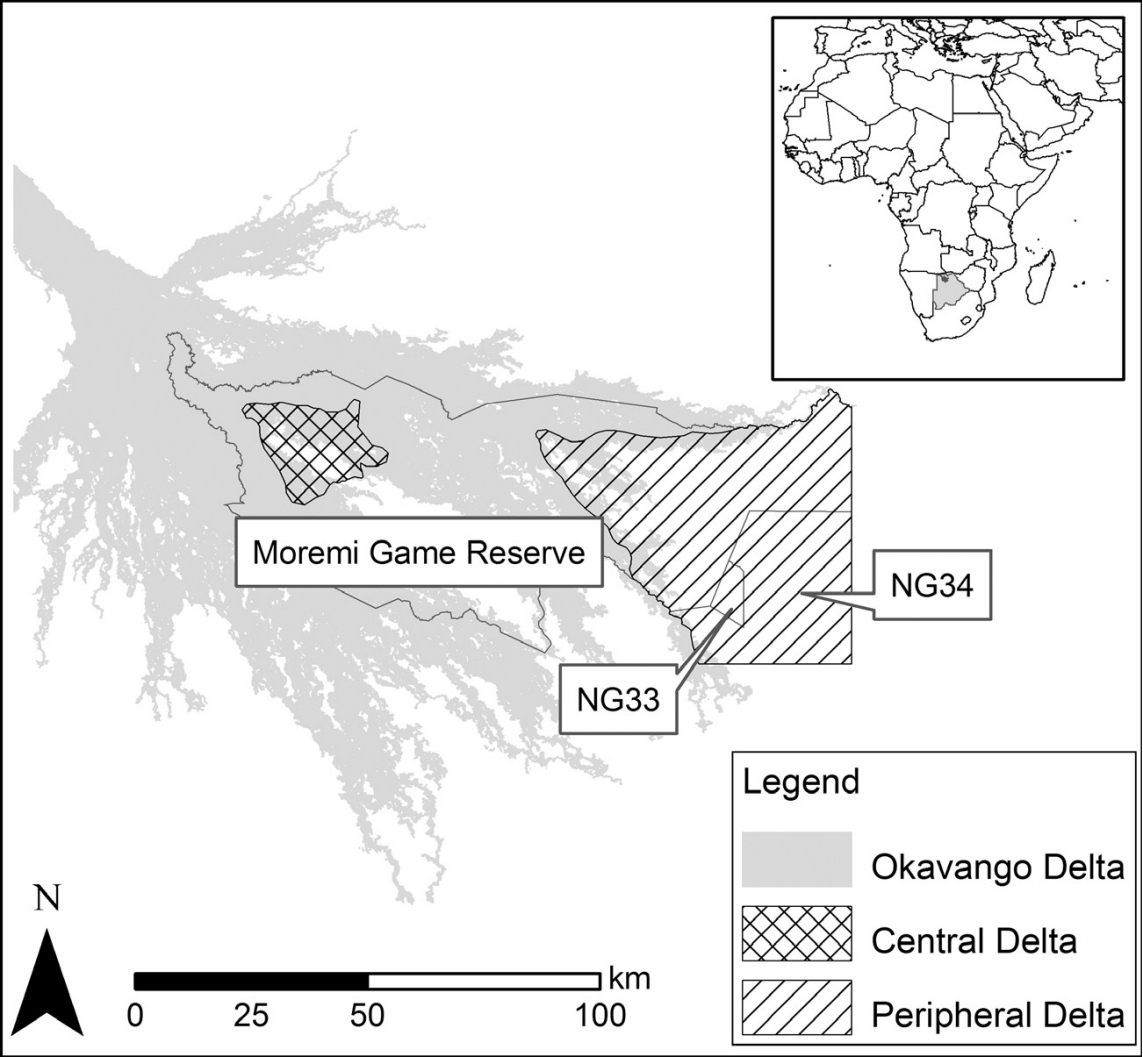
Location of Botswana (insert, shaded) and the study areas in the Okavango Delta. The Central Delta region (crosshatch shading) is located entirely in the Moremi Game Reserve, while the Peripheral Delta region (diagonal shading) includes the southeastern Moremi Game Reserve and wildlife management areas NG33 and NG34.

The study was conducted from July 2007 to October 2008 in the Moremi Game Reserve (Fig. 1) and surrounding wildlife management areas in the Okavango Delta, Botswana (Fig. 2); the study area lay between 19°05′ and 19°38′ south and 22°41′ and 23°53′ east. There were two distinct seasons. The rainy season ran from November to February, when temperatures were high. The arrival of the floods from March to June coincided with the cold part of the dry season, whereas the retreat of the flood waters from July to October coincided with the hot part of the dry season. We designated areas that flooded for more than 3 months of the year Central Delta, those that flooded for less than 3 months Peripheral Delta. There were clear differences in the sward characteristics of the two regions: forage availability was greater and more consistent in the Central Delta, with a higher availability of short stoloniferous grass communities (grazing lawns), while the Peripheral Delta had greater variability with areas of higher grass biomass but with greater intertuft distance and lower overall availability (Bartlam 2010).

### Zebra capture and radio tracking

Between April and October 2008 a total of 14 adult zebra mares, each from a different harem selected opportunistically, were fitted with GPS-capable collars (Vectronic GPS Plus 4D, Vectronic Aerospace GmbH, Berlin, Germany); seven harems were in the Central Delta and seven in the Peripheral Delta. These also had a VHF-tracking unit and activity sensor, and weighed 0.95 kg, <0.3% of the total body weight of a southern African zebra mare (mean weight 320 kg; Skinner and Smithers 1990). Mares were selected to reduce the risk of collar damage from intrasexual fighting; as zebra live in harems that include different age and sex cohorts, collaring only mares does not introduce biases due to sex- and age-specific movement patterns and/or spatial preferences.

Zebra were darted from a stationary vehicle by an experienced wildlife veterinarian and sedated with etorphine hydrochloride (M99®; Logos Agvet, Johannesburg, South Africa); mean ± SE time from darting to immobilization for all collaring and decollaring operations was 8.3 ± 4.3 min (range 2.5–22.2 min). Collars were fitted securely to the top of the zebra's neck; immobilization was then reversed with diprenorphine hydrochloride (M50-M50®; Logos Avget, Johannesburg, South Africa); mean ± SE time immobilized was 13.5 ± 6.4 min (range 3.2–36.5 min). All zebra recovered successfully; none showed any lasting effects of immobilization and/or handling, and all were observed rejoining their harems. Collars were removed at the end of the study using the same protocol. All capture and handling techniques conformed to the guidelines of the American Society of Mammalogists for the use of wild mammals in research (Sikes et al. 2011) and were approved by the University of Bristol's Ethics Committee.

The collars were programmed to take hourly fixes through the 24 h. Only 3D fixes with a DOP of less than 3.5 were used for the analyses; these constituted 97.3% of fixes. The VHF component of all collars worked 24 h a day: all zebra were tracked using radio-telemetry by air or from a vehicle at least once a month to confirm their location and, where possible, condition, and to check collar functionality.

### Home range estimation

We used a minimum of 83 consecutive days of movement data, with a mean of 172 ± 10 days and 4101 ± 244 fixes to calculate kernel density estimates (KDE) for dry season home ranges for each zebra using the Animal Movement Extension (Hooge et al. 1999) for ArcView 3.2. KDE is a contouring method (Worton 1989) and less likely to include unused landscapes in the home range estimate because it is less influenced by distant points than minimum convex polygon techniques (Powell 2000). KDE produces an utilization distribution (UD); UDs quantify an animal's relative use of space in terms of a probabilistic density function (Van Winkle 1975), removing pseudoreplication without presuming equal usage across the entire range.

Home range size was taken as the 99% KDE isopleth and calculated in km^2^ for each zebra; differences between regions were tested for significance using a nonparametric Mann–Whitney *U* test due to inequality of variances. Nearest neighbor analysis was used to test for spatial randomness within the home range using Spatial Statistics in ArcGIS 9.1, which measures the distance between each point and its nearest neighbor. If the average of these distances is less than that of the average of a hypothetical random distribution (index ratio <1), the distribution of points is considered clustered; if the average distance is greater than the average hypothetical distance (index ratio >1), the distribution is dispersed.

### Measuring landscape characteristics and resources within home ranges

The total number of habitat patches (NP), the mean shape index (measure of patch shape), and the mean index of juxtaposition and interspersion (IJI) (measure of the homogeneity of patch arrangement) (see Table 1 for full definitions) were calculated for each home range using FRAGSTATS (McGarigal et al. 2002). Proportional availability of each habitat was calculated using Spatial Analyst in ArcView 3.2. Pearson's correlation was used to test whether home range size was correlated to landscape characteristics within home ranges.

**Table 1:** Descriptions of habitat types and definitions of landscape metrics.

Landscape characteristics	Definition
Habitat types	
Floodplain	Open, seasonally flooded grasslands
Grassland	Open, shrubbed, savannah grasslands
*Acacia* woodland	Open woodland with >70% *Acacia* species
Riparian woodland	Tall open mixed woodland located in riverine or historic riverine areas
Mopane woodland	*Colophosphermum mopane* dominated woodland
Landscape metrics	
Number of habitat patches (NP)	Measure of landscape fragmentation; metric equals the number of patches in the landscape. Value ranges from 1 if one patch covers entire landscape to a maximum equal to the total number of patches
Landscape mean patch shape index (MSI)	Measure of the average patch complexity within the landscape; metric equals 1 if average patch is square, increasing without limit as shape becomes more irregular
Index of juxtaposition and interspersion (IJI)	Measure of the intermixing of different habitat patches; probabilistic metric equals 0 if some habitat types are commonly found adjacent to each other but others are rarely found adjacent to each other, ranging to 100 when all habitat types are equally adjacent to all other habitat types

Landscape metrics from FRAGSTATS (McGarigal et al. 2002).

A 200 m grid was laid over the home range to investigate how space use changed with landscape and habitat characteristics. Using the FOCAL PATCH extension (Hurvitz 2002) for ArcView 3.2, each grid cell was attributed a relative use value from the height of the UD by averaging the height of the kernel density within that cell. Every grid point was also attributed to one of five habitat classes: floodplain, grassland, *Acacia* woodland, riparian woodland, or mopane woodland. Landscape characteristics (see Table 1) were attributed to each grid point using a moving window approach. An analysis window of radius 100 m was created around each grid point and the number of patches, mean shape index, and interspersion–juxtaposition index were calculated within each circular window from the underlying habitat grid, which had a pixel resolution of 25 × 25 m.

RUFs were calculated for each zebra to investigate the relationship between space use and landscape characteristics (Marzluff et al. 2004). RUFs use a multiple regression approach to relate multiple landscape variables, in this case habitat, patch density, patch shape, and patch arrangement, to a continuous measure of use, in this case the UD height. The resulting RUF coefficients indicate the contribution of each landscape variable to the variation in the UD. To account for the spatial autocorrelation generated by natural environmental autocorrelation (Schiegg 2003) and the kernel analysis (Marzluff et al. 2004), the RUF uses a maximum likelihood procedure with a Matern correlation function (Marzluff et al. 2004). Dummy categories were created for the categorical habitat classes. The three woodland classes (*Acacia* woodland, riparian woodland, and mopane woodland) were combined into one class (woodland) and used as a reference category to which floodplain and grassland were compared. The continuous landscape characteristics were not altered.

Unstandardized RUF β coefficients were calculated for each zebra to investigate whether the effects of landscape characteristics on space use differ across the Delta. As each zebra was taken to be independent, population-level estimates were calculated by averaging RUFs from all zebra in each region, with variance calculated to include between and within zebra variance. Positive β coefficients indicate that an increase in use is associated with an increase in the characteristic, negative β coefficients indicate a decrease in use. Pearson's tests were used to check if β coefficients were significantly correlated to home range scale landscape characteristics, that is, home range area, total number of patches, mean shape complexity, mean interspersion–juxtaposition, and proportional availability of each of the five habitat classes. Relative use of available habitats (sum of UD values within habitat divided by area [km^2^] of that habitat) was tested for significance using an analysis of variance (ANOVA), with post hoc Tukey's HSD tests when appropriate.

Standardized β coefficients were used to compare the relative importance of landscape factors on the concentration of use by each zebra. They allow comparisons between the relative importance of landscape characteristics despite differences in quantifying scales:


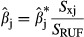


where 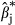 is the maximum likelihood estimate of the partial regression coefficient from the multiple regression estimate (unstandardized β), *S*_xj_ is the standard deviation of the value of resource j*,* and *S*_RUF_ is the estimate of the standard deviation of the UD values (Marzluff et al. 2004). Variance was calculated as before. The significance of individual β coefficients was determined by whether the confidence interval included zero (Zar 1999).

All means are given ±SE RUF analysis was done in R 2.8.1, using the “ruf” extension (http://csde.washington.edu/∼handcock/ruf/); other statistical analysis was done in SPSS v14.0.1.

## Results

### Home range size

**Figure 3:**
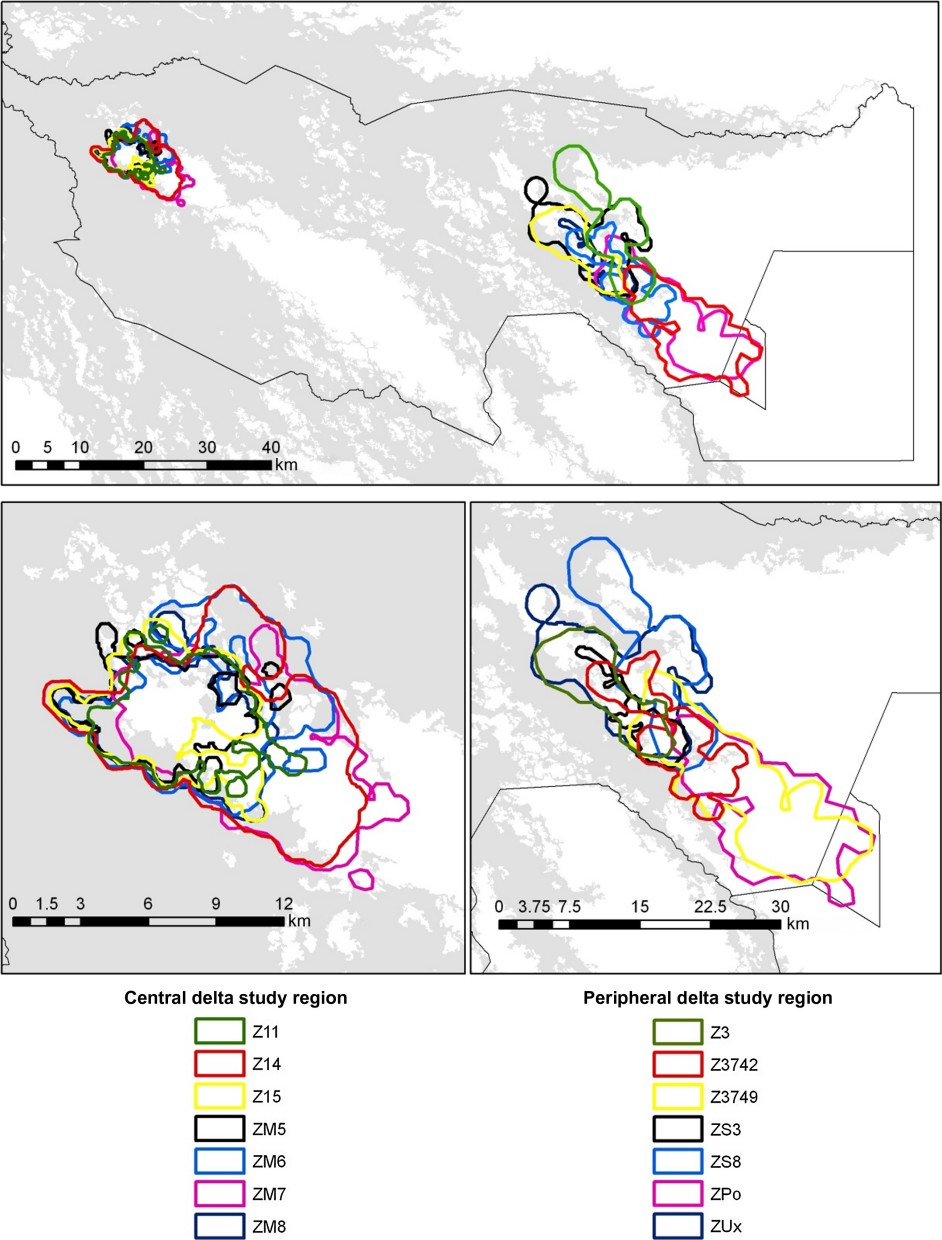
Dry season home ranges for zebra in the Central (*n* = 7) and Peripheral (*n* = 7) Delta illustrating the difference in home range size and distribution.

**Figure 4:**
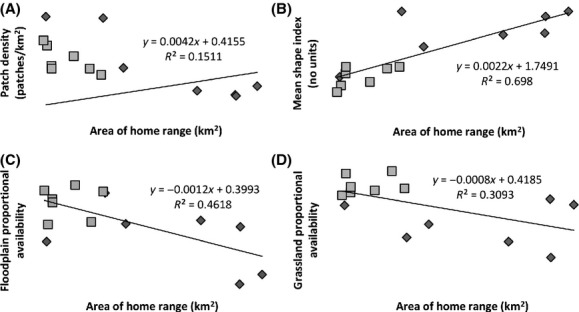
Variation in home range area with key landscape characteristics: (A) number of habitat patches per km^2^; (B) mean shape index; (C) floodplain availability; and (D) grassland availability. Squares indicate zebra in the Central Delta, diamonds zebra in the Peripheral Delta.

Home range size (Fig. 3) and within home range landscape characteristics varied by area (Table 2). Space use within all home ranges was clustered; home ranges in the Central Delta were significantly smaller compared to the Peripheral Delta (*Z* = −2.364, *P* = 0.018). Across all zebra, home range size was negatively correlated with the density of habitat patches (NP/km^2^) (*r* = −0.79, *P* < 0.001), proportional availability of floodplain (*r* = −0.680, *P* = 0.008) and grassland habitats (*r* = −0.556, *P* = 0.039), and positively correlated to the landscape mean patch shape index (MSI) (*r* = 0.835, *P* < 0.001) and proportional availability of mopane woodlands (*r* = 0.831, *P* < 0.001) (Fig. 4). No significant correlation was found between home range size and proportional availability of *Acacia* woodlands (*r* = 0.511, *P* = 0.062), riparian woodlands (*r* = −0.226, *P* = 0.438) or the IJI (*r* = −0.167, *P* = 0.569).

**Table 2:** Landscape characteristics of home ranges in both Delta regions showing differences in home range size and within home range landscape characteristics; figures are means (±SE)

					Mean proportional availability, %				
					
	Mean home range area, km^2^	Mean number of patches	Mean shape index	Mean index of interspersion and juxtaposition	Floodplain	Grassland	*Acacia* woodland	Riparian woodland	Mopane woodland
Central Delta	50.07 (7.15)	75.14 (7.59)	1.82 (0.02)	80.18 (3.94)	35.55 (2.17)	41.38 (1.31)	13.98 (0.91)	8.58 (0.55)	0.52 (0.28)
Peripheral Delta	137.52 (25.21)	151.88 (15.62)	2.08 (0.05)	80.56 (4.61)	22.30 (4.49)	27.09 (2.08)	16.48 (4.38)	14.74 (3.22)	19.36 (4.49)

### Population-level prediction of space use

In the Central Delta, areas highly utilized by zebra were associated with a greater availability of floodplains and grasslands as compared to woodland habitats, with higher densities of habitat patches, more complex patch shapes, and low juxtaposition of habitats. Highly utilized areas in the Peripheral Delta were also associated with a greater availability of floodplains and grasslands as compared to woodland habitats. However, unlike the Central Delta, high use was associated with lower densities of patches and simpler patch shapes. The interspersion of habitats did not appear to affect use (Table 3).

**Table 3:** Unstandardized resource utilization functions for zebra in the Central and Peripheral Delta, showing how land use by zebra differs between study regions

Delta region	*n*	Mean estimates of unstandardized RUF β coefficients (±SE)					

		β_Intercept_	β_Floodplain_	β_Grassland_	β_MSI_	β_IJI_	β_NP_
Central Delta	7	3.724 (0.112)	2.023 (0.122)	1.102 (0.021)	4.266 (0.101)	−0.009 (0.001)	0.997 (0.016)
Peripheral Delta	7	5.624 (0.108)	3.175 (0.051)	0.492 (0.130)	−0.221 (0.109)	−0.001 (0.001)	−0.189 (0.018)

Floodplain and grassland modeled in response to woodland.

RUF β coefficients were affected by specific home range characteristics, although these varied with Delta region. In the Central Delta the RUF β_Grassland_ was positively correlated with the mean patch shape complexity in the home range (*r* = 0.838, *P* = 0.019); the RUF β_NP_ was negatively correlated with the total NP within the home range (*r* = −0.756, *P* = 0.049) and the proportional availability of mopane woodlands (*r* = −0.877, *P* = 0.01).

In the Peripheral Delta, the RUF β_Floodplain_ was positively correlated to the proportional availability of grasslands (*r* = 0.819, *P* = 0.024) and negatively correlated to the proportional availability of mopane woodlands (*r* = −0.901, *P* = 0.06). The RUF β_MSI_ was negatively correlated to home range area (*r* = −0.764, *P* = 0.045) and positively correlated to proportional availability of riparian woodlands (*r* = 0.880, *P* = 0.009). The RUF β_IJI_ was positively correlated with proportional availability of grasslands (*r* = 0.802, *P* = 0.030) and negatively correlated to the proportional availability of mopane woodlands (*r* = 0.771, *P* = 0.043).

In the Central Delta, the relative use of habitats differed significantly from availability (*F*_4,27_ = 3.050, *P* = 0.034) (Fig. 4). Post hoc tests showed that there was a significant difference in use between floodplains, grasslands, *Acacia* woodlands, and riparian woodlands compared with mopane woodlands. There were no significant differences between availability and use in the Peripheral Delta (*F*_4,30_ = 0.505, *P* = 0.732).

### Relative influence of landscape factors on resource use

Standardized β coefficients were used to compare the relative importance of landscape factors on the concentration of use by individual zebra. The most important characteristic was not consistent among all individuals, even within the same area of the Delta (Table 4). In the Central Delta the most important factor was either the proportional availability of floodplains compared to all woodlands (*n* = 5 zebra) or the number of patches (*n* = 2 zebra); all of these were significantly greater than β = 0. In the Peripheral Delta the most important factor was either proportional availability of floodplains (*n* = 4 zebra) or the proportional availability of grasslands compared to all woodlands (*n* = 3 zebra), although only three of these β coefficients were significantly greater than 0.

**Table 4:** Standardized β RUF coefficients for zebra in each study region

	Central Delta (*n* = 7 zebra)					Peripheral Delta (*n* = 7 zebra)				
		
			Significant coefficients					Significant coefficients		
								
	Mean 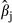	*P* (Mean 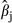 = 0)	+	−	Best predictor (no. of zebra)	Mean 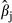	*P* (Mean 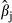 = 0)	+	−	Best predictor (no. of zebra)
β_Floodplain_	0.815	0.163	5	1	5	0.984	0.294	2	1	4
β_Grassland_	0.347	0.242	5	1		0.331	0.379	2	1	3
β_NP_	0.780	0.073	5	2	2	−0.024	0.848	1	2	
β_MSI_	0.275	0.176	5	1		−0.058	0.133	0	3	
β_IJI_	0.209	0.390	4	3		−0.052	0.505	2	2	

The table illustrates the mean standardized coefficient and the number of significant coefficients (where the 5–95% confidence intervals did not include 0) for each RUF variable in each Delta region. The highest standardized β coefficient for each zebra is the best predictor of space use.

## Discussion

Our results support the hypothesis that landscape heterogeneity and resource availability are important in determining home range size and space use in zebra. Home range size varied with the availability of key foraging habitats and habitat patch size. Differences in the availability of these characteristics across the Delta meant that zebra in the Central Delta had dry season home ranges less than half the size of those in the Peripheral Delta.

Home ranges are structured to allow efficient accumulation of key resources such as food and water (Powell 2000), and energetic expenditure is a primary constraint on home range size (Mitchell and Powell 2004). In areas with low availability or highly patchy distribution of food, animals have to move further between patches and therefore occupy larger ranges. The importance of floodplains and grasslands, habitats which have high grass availability and high grass biomass, suggests that food acquisition was the principal determinant of home range size in zebra.

Habitat patch size is an important determinant of home range size in forest-dwelling species (Saïd and Servanty 2005); decreased size correlated with increased habitat edge and therefore increased resources (Alverson et al. 1988). However, its importance in determining zebra home range size is perhaps surprising, as the highly diverse plant communities found within habitat edges (Hunter 1990) do not provide the high sward biomass zebra typically prefer. There are two possible explanations for why habitat patch size was important in determining zebra home range size; resource complementation, or patch quality (Tilman 1982; Tufto et al. 1996). Small habitat patches may be preferred by all herbivore guilds, especially if interspersion of habitats is high, as they allow animals to move more efficiently between different habitats according to their physiological demand, for example, feeding, drinking, and resting (McIvor and Odum 1988; Petit 1989), thereby decreasing home range size (Dunning et al. 1992). Alternatively, there may be a relationship between habitat patch size and patch quality, especially in the Okavango Delta, where habitat patch size is at least partially related to flooding regime, and habitat patches created by flooding are typically of higher quality than the surrounding patches. Flooding increases patchiness through gap phase succession (Watt 1947), altering species composition (Bonyongo 1999) and reversing shrub encroachment (Ramberg et al. 2006), thereby promoting the growth of homogeneous grass communities. Flooding also directly affects patch quality by locally increasing available nitrogen and phosphorous concentrations (Mubyana et al. 2003). Small floodplain patches created during seasonal flooding are therefore higher in forage quality and so are more profitable for foraging, allowing animals to consume their required nutrients more efficiently and so decrease total home range size.

Space use within zebra home ranges was clustered, indicating a nonrandom distribution. As hypothesized, open foraging habitats were associated with the heaviest relative use within a zebra's home range in both regions and were therefore the parts of the home range in which zebra spent most time. However, despite patch density being an important determinant of home range size, only zebra in the Central Delta used the high patch density areas preferentially within their home range; in the Peripheral Delta use was unrelated to patch density. If smaller patches are higher in forage quality, the differing utilization preferences between Delta areas may be due to differing foraging strategies. Optimal foraging theory states that selectivity depends on forage abundance (Pyke et al. 1977), and decreasing resource quality can make animals increasingly generalist (Bergman et al. 2001). A generalist grazing strategy is best achieved by selecting large areas of abundant forage rather than smaller areas of higher quality forage, thereby maximizing intake rate (Belovsky 1986). This may be the foraging strategy in the Peripheral Delta where sward characteristics are less favorable (Bartlam 2010) and resource quality lower (Ellery et al. 2000).

Individual variability in space use, as well as population-level differences in space use decisions, were found between the Central and Peripheral Delta. The high degree of spatial and temporal heterogeneity within the Okavango Delta may mean that no single strategy is substantially superior to others, and as such animals using very different strategies may all thrive. However, while we focussed on how resource distribution affects space use, individual decisions on space use made by free-ranging herbivores may be influenced by prior experience, and thus knowledge of the landscape (Bélisle 2005), and immediate constraints such as age, sex and breeding status (Bertrand et al. 1996) and risk of predation (Fischhoff et al. 2007). Habitat selection by herbivores alters in response to predation risk (e.g., Creel et al. 2005; Fortin et al. 2005; Fischhoff et al. 2007), and the extent of avoidance varies with environmental and social conditions. For example, zebra in a mixed savannah system were more likely to utilize dense habitat when lions were in the vicinity (Fischhoff et al. 2007). However, in a water-constrained environment, grazer distribution did not change with longer term predation pressure (Valeix et al. 2009), and predation only influenced buffalo (*Syncerus caffer*) foraging patch decisions when herd size was small (Prins 1996). While it was beyond the scope of this study to determine the comparative level of influence of other factors, their importance should not be discounted.

### Relevance to the conservation of a changing environment

The fragmentation, disturbance, and degradation of conservation areas by human encroachment, road development, water extraction, and the expansion of the fenced livestock sector is of increasing concern across Africa (Sanderson et al. 2002). Perturbations outside conservation areas are likely to be associated with changing landscape characteristics such as habitat distribution, water availability, and sward characteristics within conservation areas (Saunders et al. 1991; Fahrig 2003), all of which can influence animal space use. Seasonal flooding is the principal process currently affecting habitat patch characteristics in the Delta (Ramberg et al. 2006). Flooding extent and duration are highly variable, with seasonal inflow from the Okavango River system and total local rainfall being the key factors affecting the extent of the floods (Mendelsohn et al. 2010). The Okavango Delta is currently under threat from proposed increased water extraction from the Okavango River system and from a proposed hydroelectric dam development (Hannah et al. 1997; Mbaiwa 2004).

As flooding affects patch shape, size and quality, the direct loss of seasonal floodplains are likely to be further compounded by changes in habitat patch configuration. Our results suggest that such changes may force zebra to change their relative space use and use larger home ranges. The spatially constrained nature of the Delta, a wetland within an otherwise arid region, means that increasing home range size is likely to increase inter- and intraspecific competition for key resources and therefore lead to a decrease in population size.

Because the distribution and complexity of habitat patches are crucial in determining space use for a bulk grazer within the Okavango Delta, understanding how the factors that determine habitat complexity may change in the future is key to protecting the ecological integrity of the Delta, particularly as the Delta is also under threat from climate change (Wolski and Murray-Hudson 2008). However, the relatively short duration of data collection means that the impact of longer term climatic variation on habitat use cannot be properly established. Longer term studies are required to quantify the impact of particularly dry or wet years on the use of woodland habitats, where annual grass quality, abundance, and persistence are likely to vary considerably with wet season characteristics (Georgiadis and McNaughton 1990).
